# X-Ray Phase Contrast Tomography Reveals Early Vascular Alterations and Neuronal Loss in a Multiple Sclerosis Model

**DOI:** 10.1038/s41598-017-06251-7

**Published:** 2017-07-19

**Authors:** A. Cedola, A. Bravin, I. Bukreeva, M. Fratini, A. Pacureanu, A. Mittone, L. Massimi, P. Cloetens, P. Coan, G. Campi, R. Spanò, F. Brun, V. Grigoryev, V. Petrosino, C. Venturi, M. Mastrogiacomo, Nicole Kerlero de Rosbo, A. Uccelli

**Affiliations:** 1 Institute of Nanotechnology- CNR, Rome Unit, Rome, Italy; 2 European Synchrotron Radiation Facility, Grenoble, Cedex 9, France; 3 IRCCS Santa Lucia Foundation, Rome, Italy; 4 Ludwig-Maximilians-Universität, Faculty of Medicine and Department of Physics, München, Germany; 5 Institute of Crystallography-CNR, Monterotondo, Rome, Italy; 6 Department of Experimental Medicine, University of Genoa & Policlinico San Martino - IST Istituto Nazionale per la Ricerca sul Cancro, Genoa, Italy; 7 Moscow Engineering Physics Institute/MEPhI, Moscow, Russia; 8 Department of Neurosciences, Rehabilitation, Ophthalmology, Genetics, Maternal and Child Health Unit, University of Genoa & AOU San Martino - IST Istituto Nazionale per la Ricerca sul Cancro Genoa, Genoa, Italy; 9 Centre of Excellence for Biomedical Research, University of Genoa, Genoa, Italy

## Abstract

The degenerative effects of multiple sclerosis at the level of the vascular and neuronal networks in the central nervous system are currently the object of intensive investigation. Preclinical studies have demonstrated the efficacy of mesenchymal stem cell (MSC) therapy in experimental autoimmune encephalomyelitis (EAE), the animal model for multiple sclerosis, but the neuropathology of specific lesions in EAE and the effects of MSC treatment are under debate. Because conventional imaging techniques entail protocols that alter the tissues, limiting the reliability of the results, we have used non-invasive X-ray phase-contrast tomography to obtain an unprecedented direct 3D characterization of EAE lesions at micro-to-nano scales, with *simultaneous* imaging of the vascular and neuronal networks. We reveal EAE-mediated alterations down to the capillary network. Our findings shed light on how the disease and MSC treatment affect the tissues, and promote X-ray phase-contrast tomography as a powerful tool for studying neurovascular diseases and monitoring advanced therapies.

## Introduction

Multiple Sclerosis (MS) is an autoimmune disease of the central nervous system (CNS) associated with demyelination, axonal damage and neuronal loss resulting in neurological deficits. The targets of the immunological attack are myelin, axons and neural cells^1, 2^. In particular, autoreactive myelin-specific T and B cells infiltrate an impaired blood-brain barrier (BBB) leading to neuroinflammation and subsequent recruitment of macrophages leading to synaptic damage and ultimately neural degeneration. Current therapies target essentially the inflammation and have no significant effect in reversing CNS damage.

The use of appropriate animal models such as experimental autoimmune encephalomyelitis (EAE), induced by immunization with myelin proteins or peptides thereof, or adoptively transferred with activated myelin-specific T cells^1^, facilitates the study of disease mechanisms and the development of new therapeutic approaches for MS.

Alterations in vasculature are a central component of the demyelinating disease in EAE. The anomalous permeability of the BBB or equivalently of the blood-spinal-cord-barrier occurs two-three days before clinical disease onset^3–5^. Contrary to the capillary network, the venous vasculature remodelling in MS^6–9^ and in EAE^5, 10–13^ has been described and the open debate on vascular density is still intense. Recent studies carried out through histology techniques and/or conventional-casting micro-computed tomography on EAE induced in rats with spinal cord homogenate^13^ or induced in mice with myelin oligodendrocyte glycoprotein (MOG)^5, 12^ have described an extensive angiogenic remodelling, significantly dependent on the time course of the disease. In particular, they show an evident loss of integrity of the vessels (15 to 30 microns in diameter) during the first 4–7 days post-immunization for EAE^5, 12^. In contrast, an undeniable increase in vascular density during EAE was observed by means of vascular casting and/or immunohistochemistry, and by measuring protein levels of angiogenesis mediators, such as vascular endothelial growth factor, in CNS tissue homogenates^5, 12, 13^.

In 2014 Mori *et al*.^10^ used ultra high field-magnetic resonance imaging (MRI) to study the inflammation-mediated alterations in the lumbar spinal cord of mice with adoptive transfer EAE at the early pathological stage. While they did not find any alterations in the lumbar cord before disease onset, 5 days after the pathogenic CD4^+^ T-cell transfer, such alterations were observed at later time points, in particular during the peak phase, where at least partial occlusion of the vessels at the lumbar level was observed. MRI studies, in particular susceptibility-weighted imaging, in relapsing-remitting multiple sclerosis patients revealed a diminished visibility of the cerebral vasculature at an early stage of the disease^6, 7^ due to venous lumen shrinking, as detected on T2 weighted images^14^.

Therefore, although BBB impairment is a well-known feature of EAE, the status of the vasculature in EAE at the different stages of the disease is still unclear. Moreover, and most importantly, alterations in the capillary network are yet to be investigated. This is of particular relevance due to the recent interest generated by findings obtained with high-resolution imaging techniques revealing the existence of pathways of drainage of the interstitial fluid from the CNS parenchyma via narrow and restricted basement membrane pathways within the walls of cerebral capillaries and arteries^15–17^.

The possibility to explore the tissue by means of direct, non-destructive, highly resolved and sensitive volumetric imaging techniques, at different time points of the disease, could help shed light on these vascular alterations and how this affects the neuronal network, and get insights on fundamental CNS-specific communication pathways with the immune system which are likely to play a key role in the pathogenesis of diseases of the CNS.

At present, there is considerable interest in the innovative approach of using cell-based therapies, which may provide support for CNS repair through their neuroprotective effect^18^. In particular, preclinical studies have demonstrated the benefit of treatment with mesenchymal stem cells (MSC), a subset of adult progenitor cells with a potential regulatory effect on immune cells, in particular through modulation of T and B cell functions and inhibition of dendritic cell maturation, that also display neuroprotective features^18^. Thus, MSC ameliorate EAE, with reduced clinical severity associated with a clear decrease in demyelination and axonal loss^19, 20^. The translational development of MSC therapy would be highly impacted by a greater knowledge of its mode of action at different stages of the disease.

The techniques previously used to investigate damage in the vascular and neuronal networks in EAE-affected mice, suffer from several limitations. In particular, 2D imaging restricts spatial coverage, entails destructive sample preparation, and may lead to data misinterpretation due to the lack of information on the third dimension. MRI and conventional 3D computed tomography are limited in terms of spatial resolution and require aggressive staining and casting. In contrast, our recent work^21^ demonstrated that imaging by X-ray Phase-Contrast Tomography (XPCT) enables study of both the 3D distribution of vasculature and the single elements of the neuronal network, *without* sample sectioning and specific sample preparation. XPCT allows both the assessment of the overall 3D morphology of the sample and fine *virtual* sectioning (layers as thin as 130 nm).

Here, we exploit the ability of XPCT to generate multiscale 3D images to evaluate morphological alterations in the vascular and the neuronal networks in MOG-induced EAE in mice at two time points, disease onset and acute phase, with and without MSC treatment. We provide a direct 3D morphological description of EAE lesions at vascular level at two different length scales using: i) micro-XPCT, which gives a 3D quantitative analysis of the vasculature of the entire lumbar part of the spinal cord with spatial resolution of about 7 microns (isotropic voxel size of 3.5 micron), 1 day and 5 days after disease onset, without any staining procedure; and ii) nano-XPCT to investigate the 3D capillary network in a region of about 300 × 300 × 70 μm^3^ with an isotropic voxel size of 130 nm.

Such a multi-scale direct analysis has never been performed to dissect EAE pathology focusing on the vascular network and capillaries and addressing the effect of an innovative therapeutic strategy on this compartment.

## Results

### Alterations in the vascular network

To investigate the alterations in the vascular network caused by EAE and to evaluate how MSC treatment affects these changes, we analyzed the lumbar spinal cord region of EAE-affected mice treated or not with MSC by XPCT, 1 day and 5 days after EAE onset.

**Figure 1:**
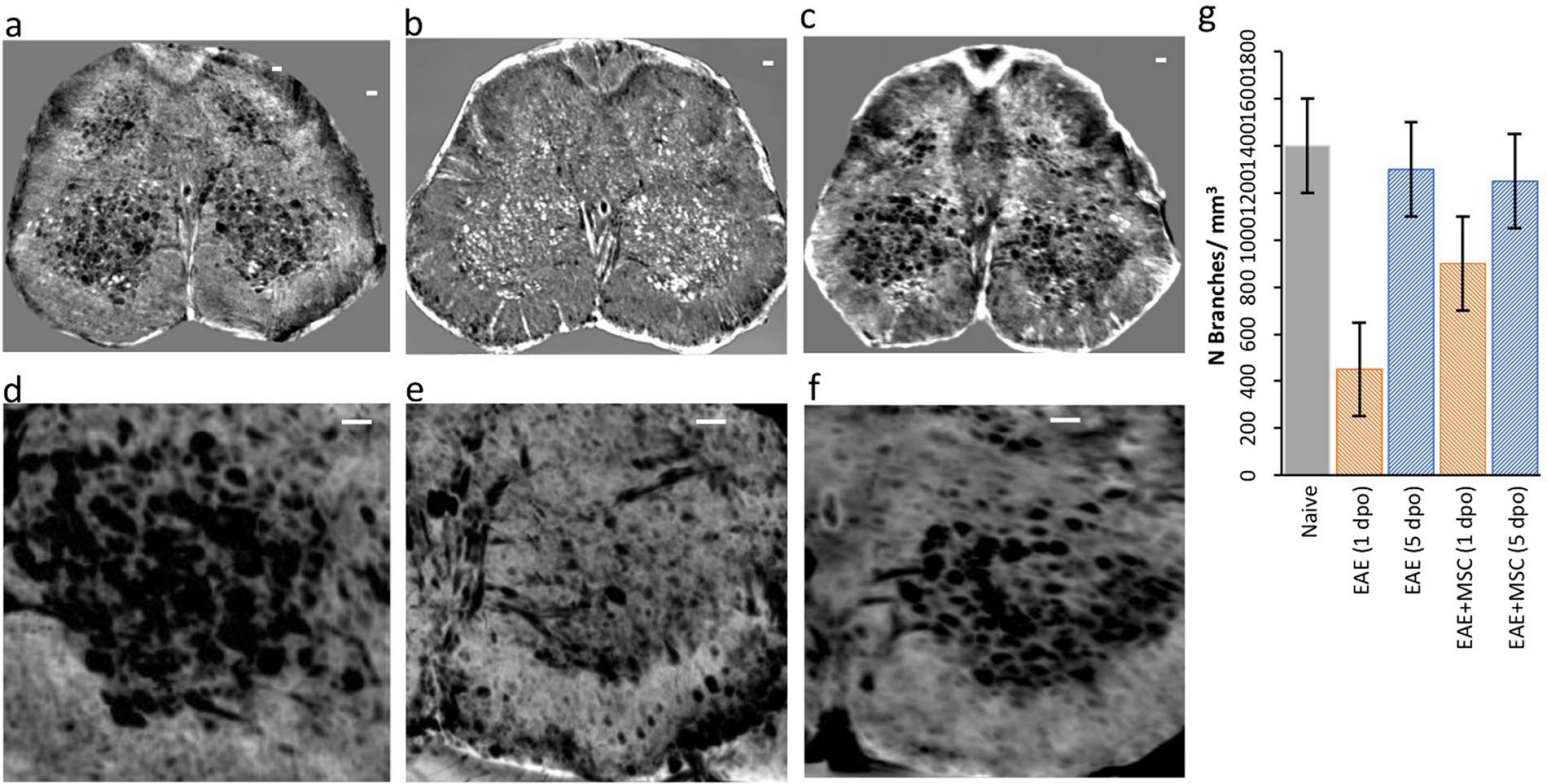
Vascular alteration at the initial phase of EAE. Axial projections of XPCT images of the spinal cord in healthy (**a**,**d**), EAE-affected (**b**,**e**), and EAE-affected MSC-treated (**c**,**f**) mice. The pixel size is 3.5 microns and the spatial resolution is 7 microns. The figures show tomography of 1.5 × 1 × 1 mm^3^ volume; (**d**–**f**) are the segmentation of the vasculature (rendered in black) of (**a**–**c**) respectively. Scale bar = 20 μm. The red arrow in (**a**) indicates the anterior spinal artery (**g**) Quantification of the number of vessel branches in lumbar spinal cord samples from naïve and EAE-affected mice at 1 dpo (red) and 5 dpo (blue), with and without MSC treatment (n = 3 per group). The data are presented as mean ± SD. The corresponding tomographic images of EAE spinal cord at 5 dpo are presented in Fig. 2S.

In Fig. 1, we compared the tomographic images of lumbar spinal cord of a healthy mouse (a), and of EAE-affected mice, untreated (b) or treated with MSC at disease onset (c); treated and untreated EAE mice were sampled one day after disease onset. The different grey-levels are proportional to different densities inside the sample. The pixel size of the images is 3.5 microns. The anterior spinal artery is clearly delineated in white in all the three samples (see the red arrow in Fig. 1a). Image segmentation (Fig. 1d–f) has been applied in order to isolate the vasculature (rendered in black) in the ventral horn of the three different samples, respectively. The micrometric spatial resolution allows the visualization of the distribution of vessels with diameters ranging from 10 to 24 microns. Vasculature density was clearly decreased in EAE affected mice compared to healthy controls (Fig. 1a–d vs 1b–e). The overall damage of the vasculature in the EAE samples resulted in a weaker detectable contrast on the image of the vessels. We also investigated vasculature alterations in mice affected with EAE 5 days post onset (dpo), i.e. during the acute inflammatory phase of the disease. Quantification of vascular alteration in three samples for each group, at both time points and with or without MSC treatment, is shown in Fig. 1g. A trend can be observed whereby vascular density in lumbar spinal cord of EAE-affected mice is clearly decreased at disease onset (EAE 1 dpo) compared to naïve mice. Such a decrease was not observed in samples from mice treated with MSC suggesting a protective effect of MSC on spinal cord vasculature (Fig. 1g). At a later stage of the disease (EAE 5 dpo), the difference in the vasculature between naïve, EAE-affected and EAE-affected MSC-treated mouse spinal cord tissues (see also Fig. 2S of the supplementary information) was not appreciable.

To address the cause of the decrease in contrast that reflects vessel alteration, we performed nano-XPCT imaging, where the presence of osmium, which binds to lipids, produces a strong contrast in cell membranes, enabling the clear detection of cells in and around the vessels (rendered in black).

**Figure 2:**
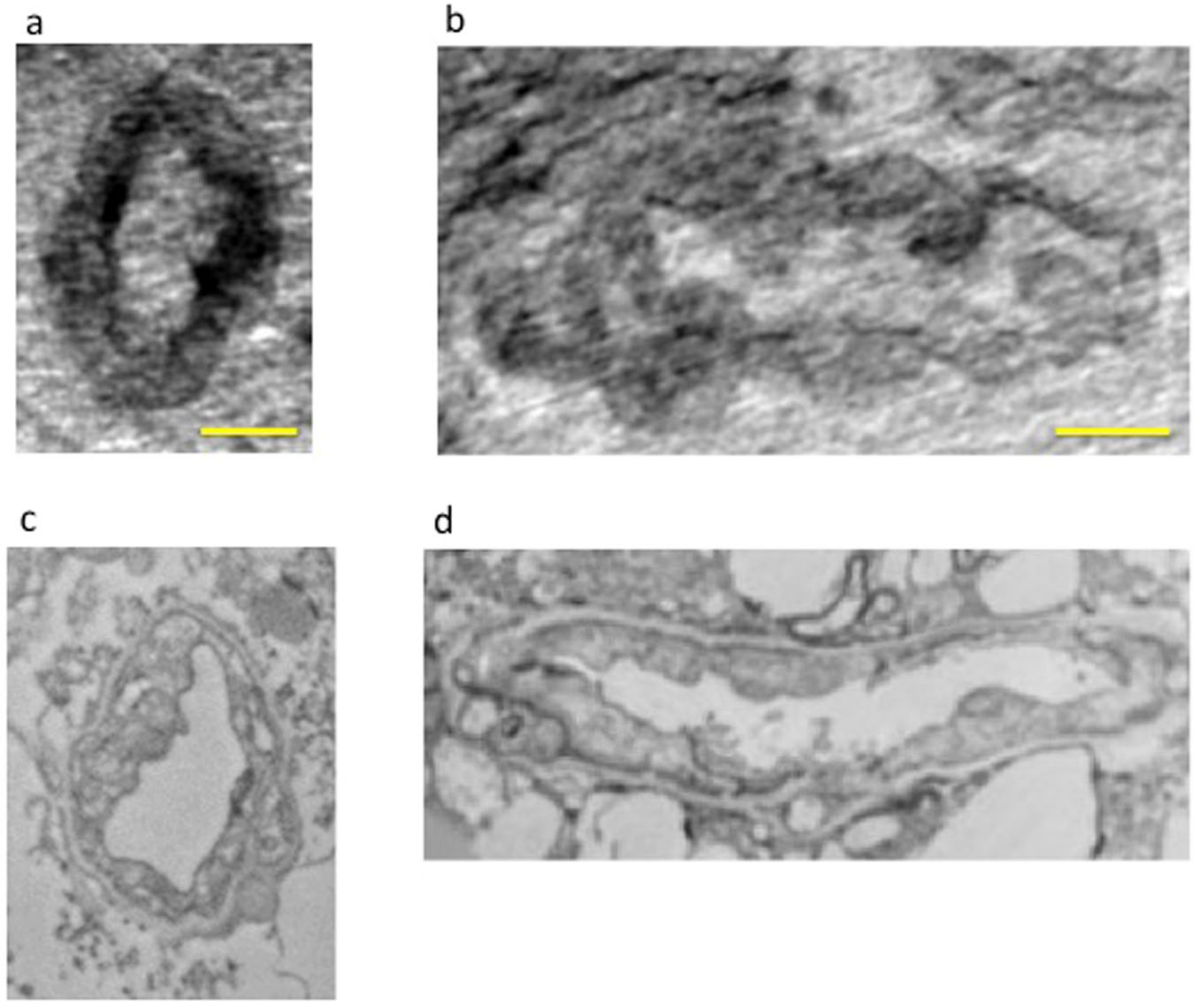
Comparison of naïve mouse CNS vessel imaging by nano-XPCT and TEM. Transversal and longitudinal sections of a capillary in naïve mouse spinal cord by nano-XPCT ((**a**,**b**) respectively) and TEM ((**c**,**d**) respectively). The thickness of the virtual slice selected by XPCT was 130 nm; the thickness of the slice analyzed by TEM was 120 nm. Scale bar = 8 μm.

**Figure 3:**
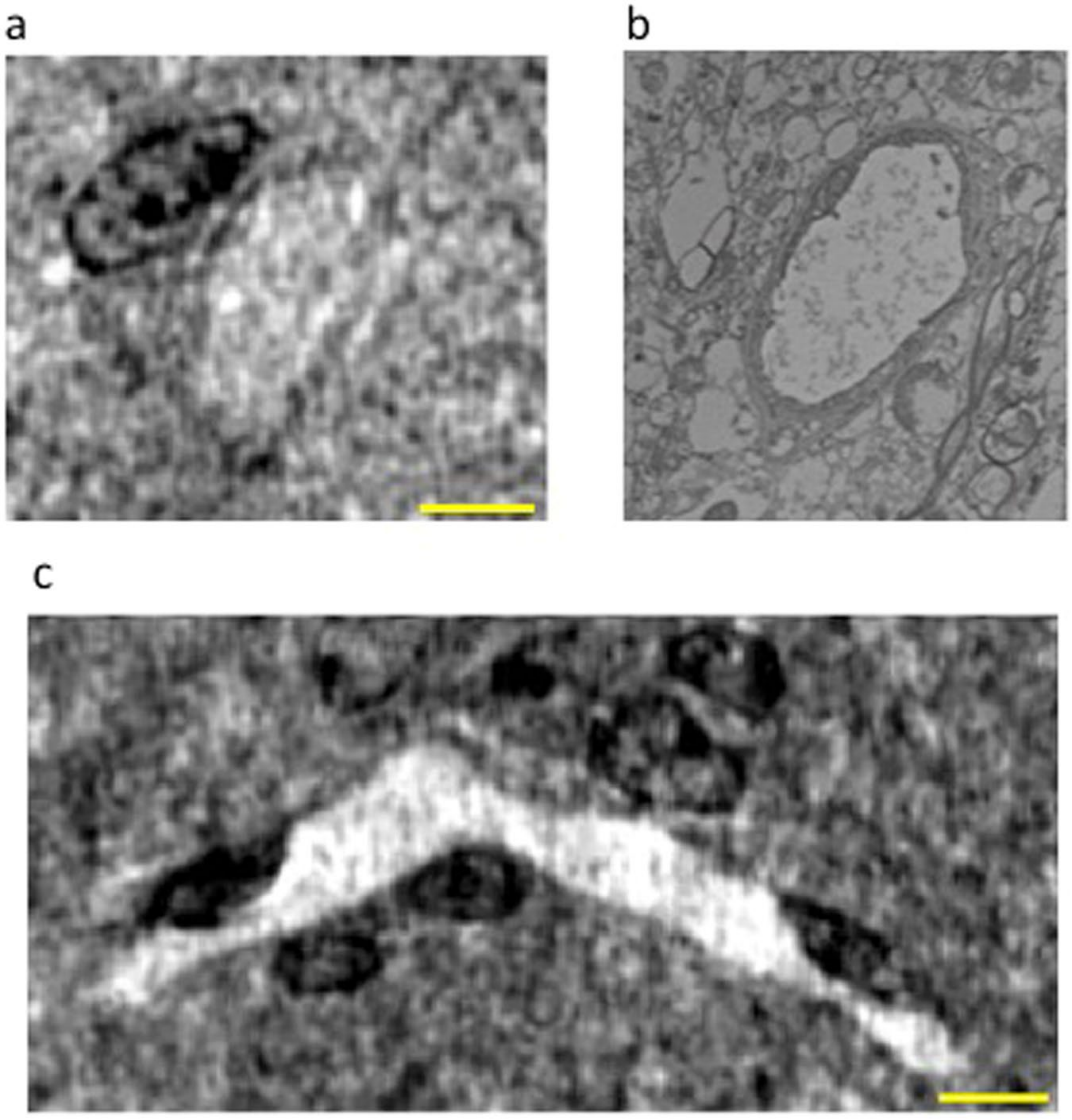
Nano-XPCT of capillaries in EAE spinal cord samples. Images of a transversal section (130 nm thick) were obtained by (**a**) XPCT and (**b**) TEM. (**c**) XPCT image of the longitudinal section of a capillary in a 20 μm-thick virtual slice. Samples were obtained at 5 dpo. Scale bar: 8 μm.

**Figure 4:**
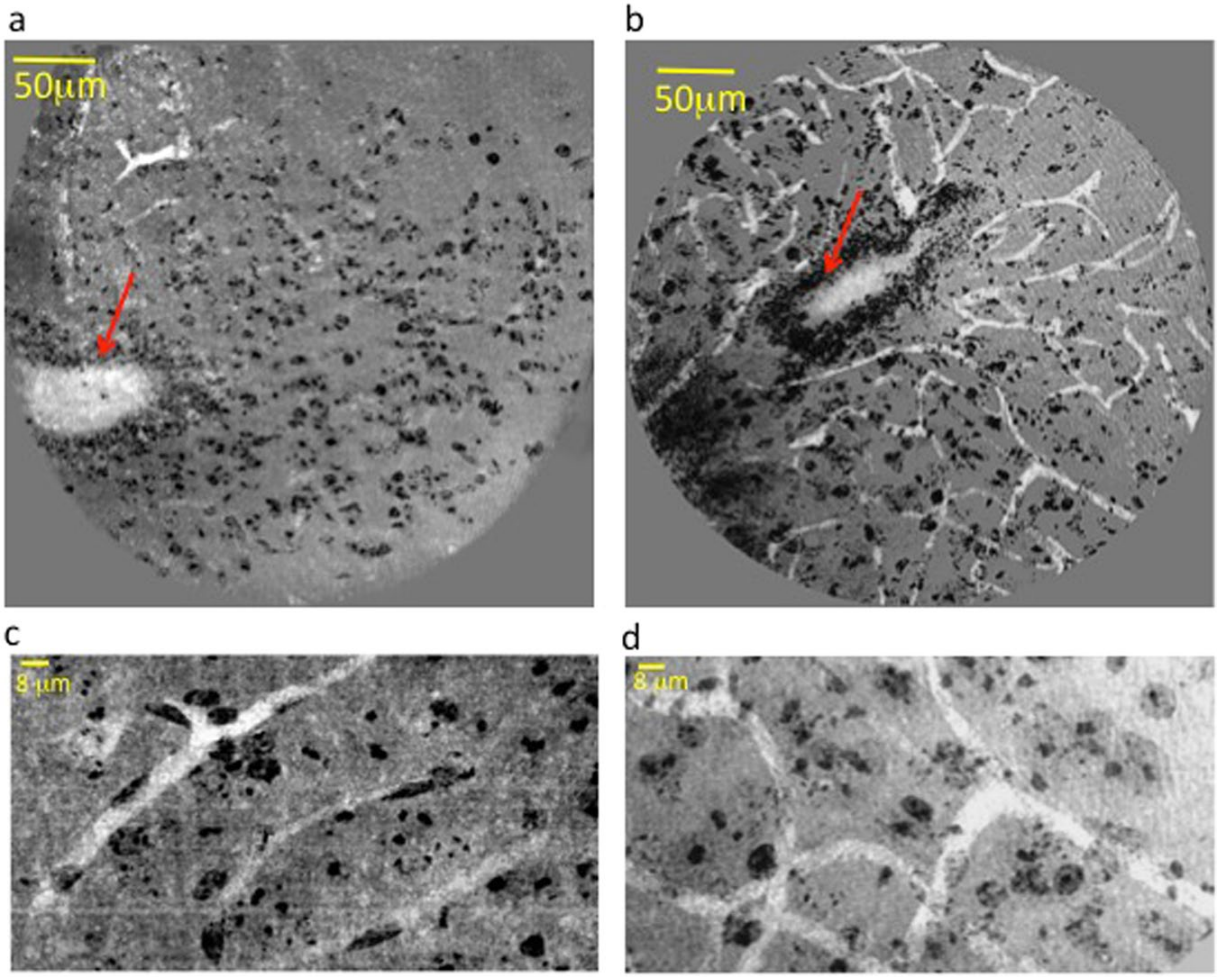
Nano-XPCT of the capillary network in spinal cord of EAE-affected mice treated or not with MSC. Axial view of the capillary network (rendered in white) and cell population (rendered in black) in untreated (**a**,**c**) and MSC-treated (**b**,**d**) EAE-affected mice. (**c**,**d**) Show magnified details of (**a**,**b**) respectively. Samples were obtained at 5 dpo.

By exploiting the nanometric spatial resolution achievable by nano-XPCT, we were able to investigate the capillary network in one naïve mouse (Fig. 2), one EAE-affected mouse (Figs 3 and 4) and one EAE-affected mouse treated with MSC (Fig. 4) with a pixel size of 130 nm. Figure 2 shows images of transversal and longitudinal sections of capillaries in a naïve spinal cord sample that were obtained through nano-XPCT (Fig. 2a,b) and TEM (Fig. 2c,d), respectively. The thickness of the nano-XPCT sections (130 nm) was computed to be in the same range as that of the TEM images (120 nm). The two different techniques deliver similar information on a 2D slice, albeit with higher spatial resolution provided by the TEM images. Figure 3 shows XPCT and TEM images of a transversal section of a capillary in a spinal cord sample from a mouse affected with EAE. The epithelial capillary wall in the EAE spinal cord sample appears thinner than in the naïve sample shown in Fig. 2. Similar observations were made for the majority of the capillaries in the EAE sample.

While TEM can only provide 2D images, XPCT can be used to get 3D information. Thus, the image in Fig. 3c, which displays the longitudinal section of a larger portion of a capillary measured in a volume of about 75 × 53 × 15 μm^3^, shows the 3D shape of the capillary and the distribution of the cells in the volume around the capillary. In particular, some groups of cells strongly marked with osmium (in black) lie over the capillary, while others are distributed in front and back with respect to the capillary.

In Fig. 4, panels a and b show the tomographic (nano-XPCT) reconstructed images of a lumbar spinal cord region spanning a volume of about 300 × 300 × 70 μm^3^ from EAE-affected mice treated or not with MSC, respectively, and sampled five days after disease onset. The analyzed regions represent similar volumes of the samples, as clearly seen according to the position of the central canal (red arrows). The overall distribution of the capillaries, imaged with approximately 130 nm spatial resolution, appears poor in EAE spinal cord (Fig. 4a). This observation contrasts with what we observed for the larger vessels, which were not appreciably altered in EAE at the same time point (see Fig. 2Sc,2Se). At higher magnification, in the image of Fig. 4c, the observed contrast between the capillaries and the parenchyma in the EAE sample is lower with respect to that observed in the MSC-treated EAE sample (Fig. 4b,d).

**Figure 5:**
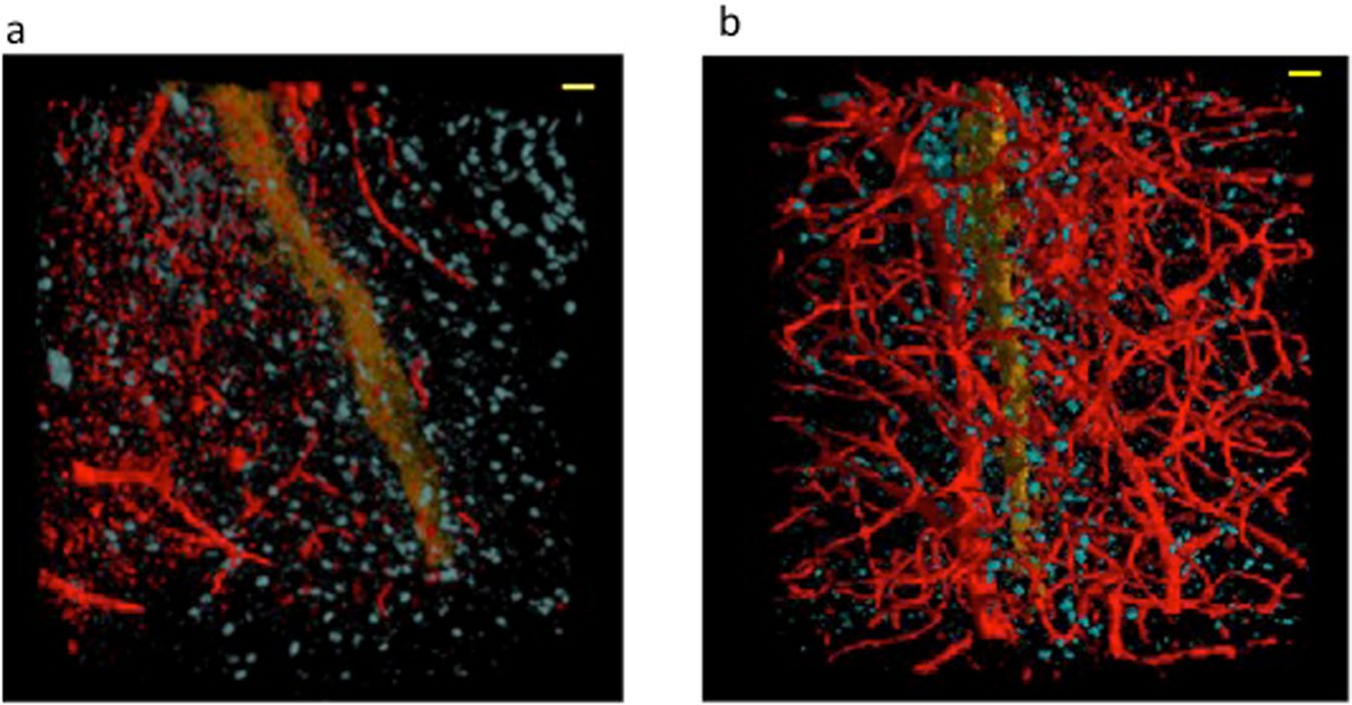
3D-rendering of the capillary network in spinal cord of EAE-affected mice treated or not with MSC. Longitudinal 3D reconstruction of the capillary network (rendered in red) and cell population (rendered in blue) in spinal cord of untreated (**a**) and MSC-treated (**b**) EAE-affected mice. The spinal canal is rendered in orange. Samples were obtained at 5 dpo. Scale bar: 8 μm. The pixel size of the nano-XPCT is 130 nm (spatial resolution ~260 nm).

Figure 5 shows the 3D rendering of the capillary network in the longitudinal view, in the EAE (Fig. 5a), and MSC-treated EAE (Fig. 5b) samples. Figure 5a shows the poor capillary network rendered in red. In Fig. 5b the capillary network appears much more ramified and dense; in the figure, we can distinguish the capillaries from the spinal canal, rendered in orange.

**Figure 6:**
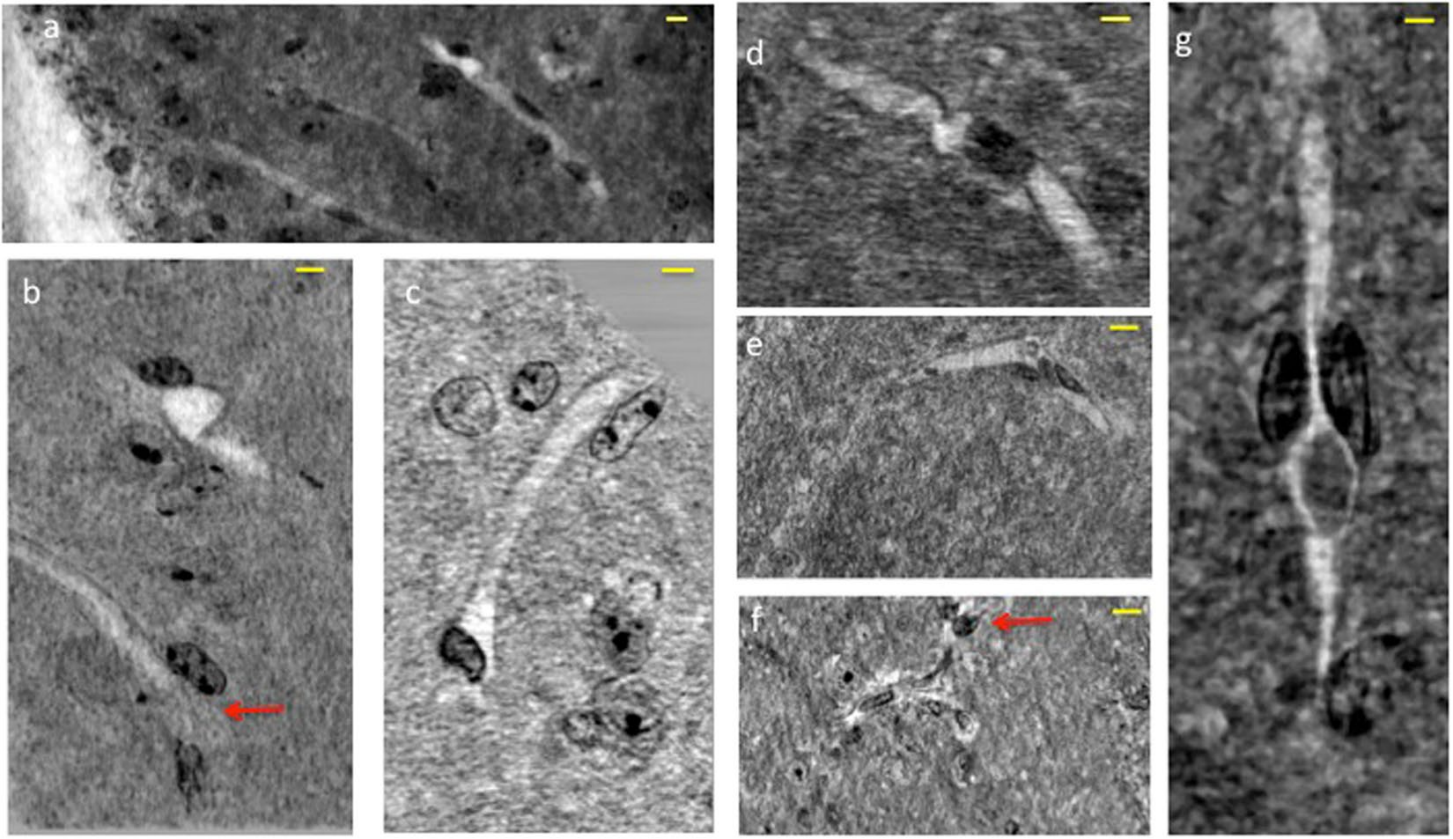
XPCT-mediated detection of capillary alterations in EAE. Details were selected in the EAE sample, in a volume of about 50 μm in thickness. This thickness is suitable to appreciate the 3D volume of the single capillary and its surroundings, an impossible task for TEM, where sections not thicker than 120 nm have to be used. The white channels are the capillaries and the black contrast comes from the osmium, which highlights the cell membranes. Scale bars: 8 μm. The pixel size of the nano-XPCT is 130 nm (spatial resolution ~260 nm).

To better understand the nature of capillary alterations in EAE-affected mice, we selected details of different sample positions, within a volume of about 50 microns in thickness. These are presented in Fig. 6 where cells, that are osmium-stained in black, can be identified inside the capillaries that appear as white channels. In particular, Fig. 6a shows the interface between the central canal (in white) and the grey matter, where the capillaries lie close to the border of the canal; at the top of Fig. 6b, we see a capillary with a discontinuous section and in weak contrast, while at the bottom one capillary bordered by two black cells appears to be open at the end (indicated by red arrow). Likely cellular obstruction of the capillary is seen frequently (Fig. 6c,d). Figure 6e shows a probable obstruction and a break at both ends. Figure 6f shows the black cells filling the white channel of the capillary (not well contrasted in the figure) and bordered by two black cells at one side (indicated by red arrow). In Fig. 6g, we can see an obstructing, non-cellular material, which appears retained through the compression of the capillary by two cells on either side. These findings suggest that nano-XPCT could allow the identification of capillary alterations in the spinal cord of mice with EAE, that result in decreased contrast of the vessels.

### Alterations in the neuronal network

**Figure 7:**
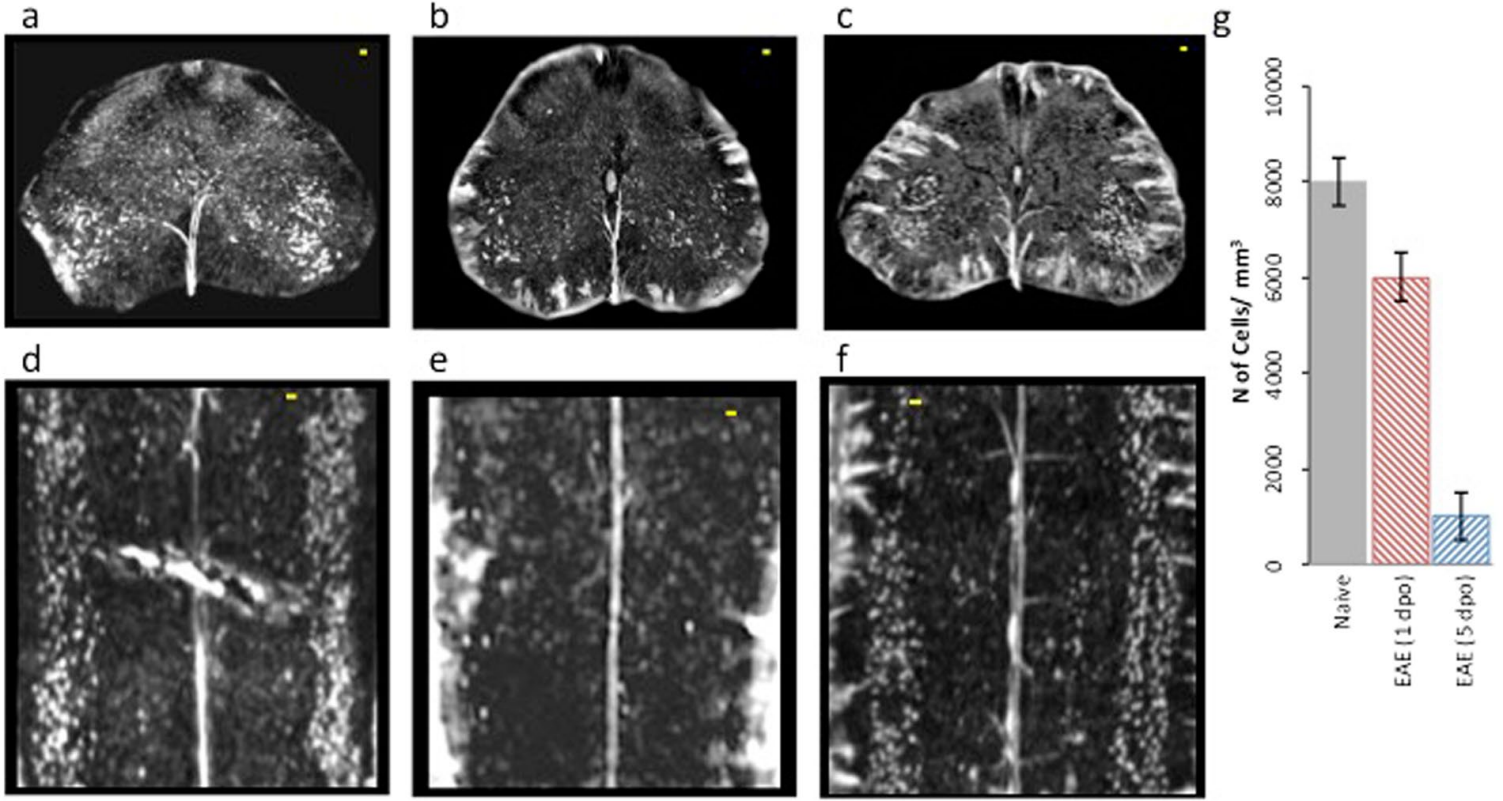
Effect of MSC treatment on the alteration of the neuronal network in EAE. Representative axial (**a**–**c**) and longitudinal (**d**–**f**) XPCT images of spinal cord samples from naïve (**a**,**d**), and untreated (**b**,**e**) or MSC-treated (**c**,**f**) EAE-affected mice at 5 dpo. Neurons appear as white spots. (**g**) Quantification of the number of cells for naïve, and untreated EAE at 1 and 5 dpo. These data were obtained on the same set of samples used to assess vascular alteration in Fig. 1g. Scale bar: 20 μm. The pixel size is 3.5 microns and the spatial resolution is 7 microns.

Next, we used XPCT to investigate changes in the neural component of the spinal cord of EAE-affected mice, 1 day and 5 days after EAE onset. Through micro-XPCT we compared the moto-neuron distribution in the ventral horn of the spinal cord of the same set of samples used to analyze the vascular alteration shown in Fig. 1. To identify the motor neurons, we have exploited an algorithm that we recently developed^22^. Built on anatomical basis and on the typical moto-neuron size, this algorithm is validated by a comparison with the quantitative immunohistochemical analysis of CNS tissue sections developed with SMI-32 antibody, which stains moto-neuronal cell bodies. Figure 7 shows the results obtained for spinal cord samples of naïve (Fig. 7a,d), EAE-affected untreated (Fig. 7b,e), or MSC-treated (Fig. 7c,f) mice, at 5 dpo. Axial views (Fig. 7a–c) show a drastic decrease in contrast of neurons (white spots) in the EAE sample (Fig. 7b), supported by the histological analysis (see Fig. 3S), as compared to the naïve sample (Fig. 7a). Such decrease of neurons was not detected in the spinal cord of the EAE-affected mice that were treated on the day of clinical onset with MSC (Fig. 7c), as supported also by the histological analysis (see Fig. 3S). The longitudinal views (Fig. 7d–f) very clearly show the dramatic loss of detectable contrast of the motor neurons in EAE (Fig. 7e) and the protective effect exerted by MSC on motor neurons (Fig. 7f). In order to quantitatively assess changes in neuronal density observed in Fig. 7a–f, we measured the number of motor neurons in the three cases by means of our recently developed algorithm^22^. Figure 7g shows the overall results obtained in the naïve and in the EAE samples at the two different time-points. These representative results show a clear trend towards a decrease in cell numbers as early as one day after disease onset, followed by a dramatic decrease five days after disease onset.

**Figure 8:**
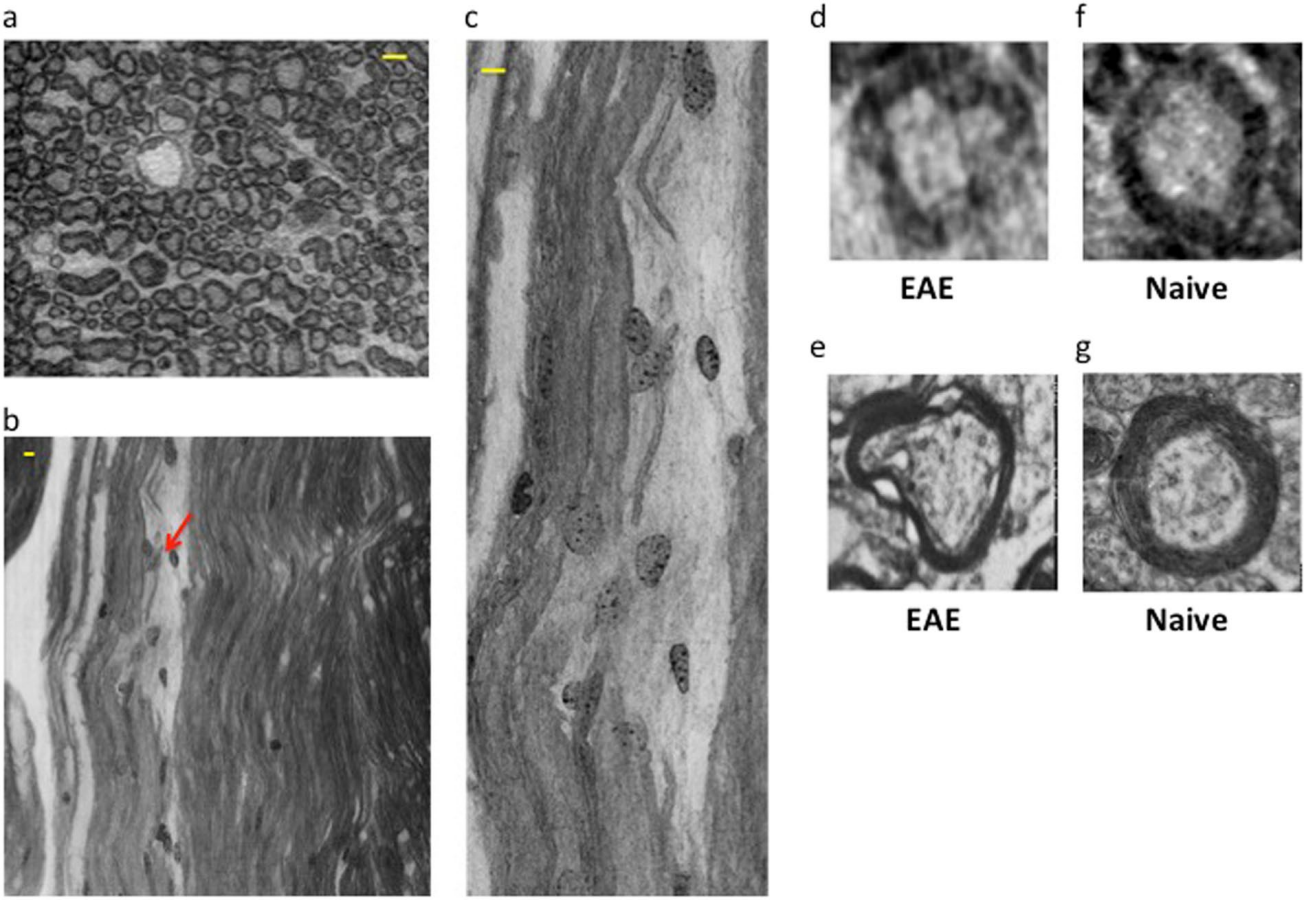
Nano-XPCT of myelin damage in EAE. (**a**,**b**) Show the axial and longitudinal views of the white-grey matter interface in the spinal cord of EAE-affected mouse sample (5 dpo). Osmium-stained myelin (black) can be seen around axon fibers. (**c**) is a magnification of **b** in the region where invading cells can be seen associated with damaged myelin. d-g show the axial views of a myelinated axon by XPCT (upper panels) and TEM (lower panels) in EAE-affected (**d**,**e**) and naïve (**f**,**g**) samples. Scale bars 8 μm. The pixel size of the nano-XPCT is 130 nm (spatial resolution ~260 nm).

In MS and EAE, demyelination is a hallmark of disease. We therefore investigated demyelination through nano-XPCT of the same osmium-treated EAE samples where myelin appears in black. Axial (Fig. 8a) and longitudinal (Fig. 8b) views of EAE spinal cord demonstrate disruption of myelin integrity in EAE samples, with numerous cells associated with damaged myelin (indicated with red arrow). Figure 8c is a magnification of a region where the cells appear closely associated to damaged myelin. In Fig. 8d,e, we present sections of an axon at a higher magnification, where the swelling of the myelin sheath in the EAE sample can be detected as gaps of weaker contrast by nano-XPCT and by TEM, respectively. Figure 8f,g show the corresponding nano-XPCT (Fig. 8f) and TEM (Fig. 8g) images of healthy axons in naïve spinal cord.

## Discussion

X-ray Phase-Contrast multiscale-Tomography allows for the 3D simultaneous investigation of the neuronal and vascular microanatomy without any invasive sample preparation or sectioning, which characterize the conventional techniques and limit the reliability of the results.

In this study, 3-D analysis of vascular networks in EAE by micro-XPCT showed a decrease in vessel density at disease onset as compared to healthy mice, which was not observed in EAE-affected mice treated with MSC. While the effect of MSC on vascular remodeling in EAE has not been investigated, vascular alterations have been studied in MS and EAE by 2-D immunohistochemical analysis of tissue slices and/or vascular casting^12, 13^. In contrast to the results obtained with 3-D XPCT analysis, these techniques demonstrated features consistent with angiogenesis and endothelial cell proliferation in both MS and EAE^5, 8, 12, 13^. Such vascular remodeling appeared to be an early process in EAE induced with MOG, as increased vessel areas and endothelial cell proliferation were detected as early as 4 days post disease induction, that is in the pre-symptomatic phase^5^. However, in a different EAE model induced in rats with guinea pig spinal cord homogenate, an increased density in small blood vessels was observed at a relapsing, later stage of the disease^13^. It has been suggested that this abnormal vascular remodeling response in EAE leads to the formation of leaky angiogenic blood vessels in the CNS concordant with the well-known loss of BBB integrity from early EAE stages^8, 12, 13^.

While these reports appear to contradict our XPCT findings, we have also observed increased staining for the endothelial cell marker CD31, indicative of angiogenesis, through immunohistochemistry of EAE tissue obtained at 5 dpo (data shown in Supplementary Fig. 3S). We note that 3-D analysis of the vascular networks in MS and EAE by MRI yielded apparently different data, which are in line with our XPCT results. Thus, Sinneker *et al*., using 7 Tesla MRI, revealed vascular alterations from early stages of MS, with reduced visibility of periventricular veins^7^. Those findings corroborated the results of two earlier 3 Tesla MRI investigations^6, 23^. Such studies, however, have not been performed in EAE. At this stage, we can only speculate on the origin of the reduced vascular contrast we observed in EAE, which apparently does not occur, or at least not to the same extent, in MSC-treated EAE mice. One possible origin is the increased fragility of vessels in EAE: they could have collapsed during the fixation of the tissue so that the contrast between vessels and the surrounding parenchyma is no longer apparent. Another possibility is the potential occlusion of the vessels, such as we have seen at nano-XPCT. In this context, it is interesting to note that a decreased cerebral blood flow has been demonstrated by perfusion-weighted MRI in grey and white matter of MS patients^24–26^. It is highly significant that the reduced contrast of the vessels is less intense in MSC-treated mice; in this context, we have seen in immunohistochemistry studies that the BBB is far less compromised in MSC-treated than in untreated EAE mice, with intact tight junctions and fewer leaky vessels (data not shown), suggesting that the vessels are less fragile in MSC-treated EAE mice.

Alteration of the capillary network in EAE has not been described before, nor has the alteration of the 10–24 micron vessels ever been evidenced without contrast agent. Our results strongly indicate a trend in alteration of the 10–24 micron vessels at the early stage of the disease and our 3D technique applied for the first time to the study of a neurodegenerative disease, has allowed us to image possible occlusions in the capillaries.

Thus, the XPCT has made it possible to investigate the vascular and neuronal alterations simultaneously during EAE. It corroborates findings by 2D techniques showing that MSC administration reduces vascular alteration of vessels as well as the damage to myelin and neurons in EAE, and provides a more informative 3D vision of the situation. In particular, our data support the findings of Vogt *et al*. who demonstrated, through electrophysiological and morphological analyses, a massive loss of lower motor neurons in MS patients and in mouse MOG-EAE from early disease stages, with evidence of apoptotic neuronal death^27^. Several other studies have also reported neuronal apoptosis and/or abnormalities in the same and other models of EAE^28, 29^, as we also observed (Fig. 4S).

The data obtained here by XPCT suggest that the angiogenesis detected at the acute phase of EAE by other means is in fact not efficient, and that the massive loss of neurons observed corroborates the demonstration of apoptotic pyknotic neurons observed by 2D techniques.

Our work shows the potentiality of XPCT to provide complementary information on the alterations of vascular and neuronal networks. To our knowledge this technique has never been applied before to similar investigations on MS or EAE models. It allows us to derive crucial information not obtainable with other techniques, usable for the study of disease evolution, as well as for the optimization/monitoring of advanced new therapies.

Moreover, the recent interest generated by findings revealing drainage of the interstitial fluid from the CNS parenchyma via narrow and restricted basement membrane pathways within the walls of cerebral capillaries and arteries makes the detection and monitoring of the capillary network of particular relevance.

This work paves the way to future *in vivo* applications of XPCT towards the investigation of different neurodegenerative diseases and the monitoring of therapies.

## Methods

### EAE induction and treatment with MSC

Female C57BL/6 J mice, 6 to 8 weeks old, weighing 18.5 ± 1.5 g, were purchased from Harlan Italy. All animals were housed in pathogen-free conditions and treated according to the Italian and European guidelines (Decreto Legislativo 4 marzo 2014, n. 26, legislative transposition of Directive 2010/63/EU of the European Parliament and of the Council of 22 September 2010 on the protection of animals used for scientific purposes), with food and water ad libitum. The research protocol was approved by the Ethical Committee for Animal Experimentation of the University of Genoa (Prot. 319). After a one-week period of adaptation to the animal house, mice were immunized as described before ref. 30 by subcutaneous injection at two sites in the flank with an emulsion of 200 μg myelin oligodendrocyte glycoprotein (MOG) peptide 35–55 (Espikem) in incomplete Freund adjuvant (IFA; Difco) containing 300 μg/ml Mycobacterium tuberculosis (strain H37Ra; Difco). Mice were injected in the tail vein with 400 ng pertussis toxin (Sigma-Aldrich) immediately and 48 h after immunization. The mice were scored daily for clinical manifestations of EAE on a scale of 0 to 5^30^. Where appropriate, mice were treated on the day of clinical onset by injection in the tail vein of mouse MSC (1 × 10^6^ MSC in PBS) that had been prepared and characterized as previously described^19^.

### Preparation of CNS samples for TEM and nano-XPCT

Mice under deep anesthesia with ketamine/xylazine cocktail (90 mg and 4.5 mg/kg, respectively; intraperitoneal injection) were transcardially perfused with heparin and physiological solution. The spinal cords were removed and cut according to specific regions (cervical, thoracic, and lumbar) that were placed in 0.1 M cacodylate buffer pH 7.2, containing 2.5% glutaraldehyde for 3 hours at room temperature. Spinal cord samples were postfixed in osmium tetroxide (1% in 0.1 M cacodylate buffer, pH 7.2; 1 hour) and uranyl acetate (1% in water; 1 hour). Samples were then dehydrated through a graded ethanol series (70/95/100%), put in propylene oxide and embedded in resin (Poly-Bed;Polysciences, Inc., Warrington, PA) at 42 °C overnight and for 2 days at 60 °C. Samples were further processed for nano-XPCT or TEM. For TEM analysis, ultrathin sections (50 nm) generated with an ultramicrotome (Leica Ultracut UCT) and stained with uranyl acetate (5% in 25% ethanol) and lead citrate (0.28% in water) were analyzed with a transmission electron microscope (model CM10; Philips).

### Statistical analysis

XPCT- We analyzed 3 samples for each of the two following typologies and considered the medium values in the number of branches with the corresponding standard deviation:

1. Naïve, EAE and EAE with MSC at 1 day from the onset.
2. Naïve, EAE and EAE with MSC at 5 days from the onset.

### X-ray Phase Contrast Tomography

Classical X-ray radiography and tomography are based on absorption and are well-known tools for imaging the internal structure of thick objects with hard X-rays. In the study of low-absorption materials (like biomedical samples), as in high resolution investigations and in particular when details with small differences in density must be detected (as in the structure of the grey and white matter of the spinal cord), the significant degree of attenuation in the sample makes producing a detectable contrast very difficult. In these cases, a better contrast can be achieved by imaging the phase modulation induced by an object in a coherent or partially coherent beam^31^.

Additionally, tomography provides the additional benefit of discriminating the different depths within the sample and providing a 3D sample reconstruction. Several experimental approaches exist for detecting X-ray phase contrast. A simple yet effective phase-contrast method for hard X-rays is based on in-line imaging after free-space propagation. When X-rays illuminate the sample, variations in optical-path length produce slight local deviations (refraction) of the X-ray beam from its original path. Generally, in absorption radiography the detector is placed close enough to the sample that these variations go unnoticed. On the contrary, when a free-space propagation distance is allowed between sample and detector, the recorded image contains the refraction information in the form of interference fringes, whose detectability depends on the coherence of the X-ray beam. Synchrotron-radiation (SR) X-ray sources provide high-photon flux X-ray beams, with a high degree of spatial coherence and allow for the possibility of providing monochromatic beams. The high quality of the images helps optimise the algorithms used for image analysis and the three-dimensional reconstruction.

The XPCT experiments were carried out at ID17 beamline at ESRF. The monochromatic incident X-ray energy was 30 keV. The sample was set at a distance of 2.3 meters from the CCD camera with a pixel size of 3.5 µm. As the sample was larger than the horizontal field of view of the CCD, projections were acquired using the half-acquisition method^32^. The tomography was produced by means of 2000 projections covering a total angle range of 360° and with an acquisition time of 1 s per point. The spatial resolution, defined as the minimum distance between two objects in order to resolve them, is approximately twice the pixel size, i.e. 7 microns.

The phase retrieval algorithm has been applied to the projections of the tomographic scans using a modified version of the ANKAphase code^33, 34^. In Fig. 1S of the Supplementary Information the difference between an untreated image and an image treated with the algorithm is shown.

The different electron densities of the tissues were rendered as grey-levels in the phase tomograms images. The image analysis and the image segmentation to independently display the different tissues, have been performed using both free software (Volview, ImageJ) and software developed in-house (Matlab routines). The quantification of the number of vessel branches^35^ and number of moto-neurons^22^ was performed by using a dedicated approach. Briefly, an intensity-based segmentation has been used to extract voxels assigned to vessels thanks to the analysis of the image histogram, while we distinguished the motor neurons exploiting an algorithm based on an anatomical basis and on the typical size. The correspondence with the neuron-specific histology has demonstrated the validity of the algorithm.

### Nano X-ray Phase Contrast Tomography

The nano-XPCT experiments were carried out at the new Nano-Imaging ID16A beamline of the ESRF (Pacureanu *et al*., submitted). This new beamline offers a unique combination of nanofocus (~30 nm) and a very high photon flux (up to 10^12^ photons/s at ΔE/E ~ 1%). A pair of multilayer coated Kirkpatrick-Baez (KB) optics was used to focus the X-rays at 17 keV. In this case, sufficient coherence is needed as well to obtain a focus that is small enough and compatible with the spatial resolution.

The sample is put in the divergent beam downstream of the focus to produce magnified phase contrast images. The magnifying geometry of the cone beam allows to overcome the spatial resolution limit of the detector and obtain a resolution similar to the size of the focus. The projection geometry also allows zooming into specific regions of a large sample by combining scans with different magnifications and fields of view^36, 37^. Due to free space propagation of the X-ray beam the contrast in the images is dominated by phase contrast, related to the real part of the complex refractive index, which is on its turn determined by the electron density of the material. By measuring the Fresnel diffraction patterns at different effective propagation distances, the phase maps of the sample can be retrieved via holographic reconstruction, the so-called phase retrieval procedure^38^ implemented using GNU Octave software. Magnified radiographs were recorded onto an X-ray detector using a FReLoN charged-coupled device (CCD) with a 2048 × 2048 binned pixels array and a 3 µm pixel size. For one tomography scan, 1500 projections were acquired with 0.32 s exposure time and 130 nm pixel size, i.e. a magnification of 23X. Tomography scans at four different focus to sample distances were acquired to complete one holotomography scan. The 2D phase maps retrieved from the angular projections were used as input for a tomographic reconstruction based on the filtered back projection algorithm method (ESRF PyHST software package). The reconstructed 3D volumes are proportional to the changes in electron density of the sample. The pixel size of 130 nm is large compared to the ultimate resolution of the ID16A setup, which is roughly 40 nm as limited by the 30 nm focus size and the sub-20 nm mechanical errors. Therefore the real spatial resolution is also in this case roughly two times the pixel size or 260 nm.

### Assessment of Radiation Damage

For XPCT, the samples were included in *agar agar*, which keeps them hydrated and prevents both radiation damage and the shift of the samples during the measurement. Similar comments apply for nano-XPCT, where the samples where embedded in Epon. In the case of nanotomography, projections are re-acquired at specific angles after the tomography scan. This allows evaluating the sample drift and possible changes due to radiation damage. At this resolution, no such changes were observed. After all X-ray measurements, none of the samples showed any visual sign of radiation damage.

### Data availability

The authors declare that the data supporting the findings of this study are available from the corresponding author on reasonable request.

## Electronic supplementary material

Supplementary Information
