# Supplementary material for: X-Ray Phase Contrast Tomography Reveals Early Vascular Alterations and Neuronal Loss in a Multiple Sclerosis Model
Source: Sci Rep. 2017 Jul 19;7:5890. doi: 10.1038/s41598-017-06251-7 (PMC5517657; doi:10.1038/s41598-017-06251-7)
Supplement: Supplementary file 1 — Supplementary Information [file 41598_2017_6251_MOESM1_ESM.pdf]

X-RAY PHASE CONTRAST TOMOGRAPHY REVEALS EARLY VASCULAR ALTERATIONS AND NEURONAL LOSS IN A MULTIPLE SCLEROSIS MODEL

A. Cedola[1*] A. Bravin[2] I. Bukreeva[1] M. Fratini[1,3] A. Pacureanu[2] A. Mittone[2] L. Massimi[1] P. Cloetens[2] P. Coan[2,4] G. Campi[5] R. Spanò[6] F. Brun [1] V. Grigoryev[7] V. Petrosino[8] C. Venturi[8] M. Mastrogiacomo[6] N. Kerlero de Rosbo[8] A. Uccelli[8,9]

[1] Institute of Nanotechnology- CNR, Rome Unit, Rome, Italy

[2]European Synchrotron Radiation Facility, Grenoble, Cedex 9, France

[3] IRCCS Santa Lucia Foundation, Rome, Italy

[4]Ludwig-Maximilians-Universität, Faculty of Medicine and Department of Physics, München, Germany

[5]Institute of Crystallography-CNR, Monterotondo, Rome, Italy

[6]Department of Experimental Medicine, University of Genova & AUO San Martino - IST Istituto Nazionale per la Ricerca sul Cancro, Genova, Italy

[7]Moscow Engineering Physics Institute / MEPhI, Moscow, Russia

[8] Department of Neurosciences, Rehabilitation, Ophthalmology, Genetics, Maternal and Child Health Unit, University of Genoa & AOU San Martino - IST Istituto Nazionale per la Ricerca sul Cancro Genoa, Italy

[9] Centre of Excellence for Biomedical Research, University of Genoa, Italy

PHASE RETRIEVAL ALGORITHM

X-ray Phase Contrast Tomography

The phase retrieval algorithm of the images acquired with X-ray Phase Contrast Tomography has been applied to the projections of the tomographic scans using a modified version of the ANKAphase code, well described in the Reference 1 and 2. Figure 1S shows the difference between an untreated image and an image treated with the algorithm.

X-ray Phase Contrast nano-Tomography

The images recorded at different distances have different magnification. The value of the magnification at the different distances is well known and calibrated: M = (z1+z2)/z1, where z1 is the focus-to-sample distance and z2 is the sample-to-detector distance. Therefore, the effective pixel size at a given distance is given by:

$$\frac{pixel\ size\,\det ector}{M}$$

To register the images, they are first resampled (bi-cubic interpolation) to bring them to the same magnification as the first distance with the smallest effective pixel size. Subsequently each distance is aligned with respect to the previous one using cross-correlation techniques. The required translations are calculated every hundred projections and the values for the intermediate projections are determined by polynomial interpolation.

In the measured images both absorption and phase contribute to the contrast, but phase contrast still dominates due to the relatively large propagation distances in the projection geometry. The phase retrieval algorithm handles the

two different contributions and the final reconstructions are proportional to the refractive index decrement, hence the changes in electron density. To a good approximation the final grey level scales linearly with the mass density inside the sample.

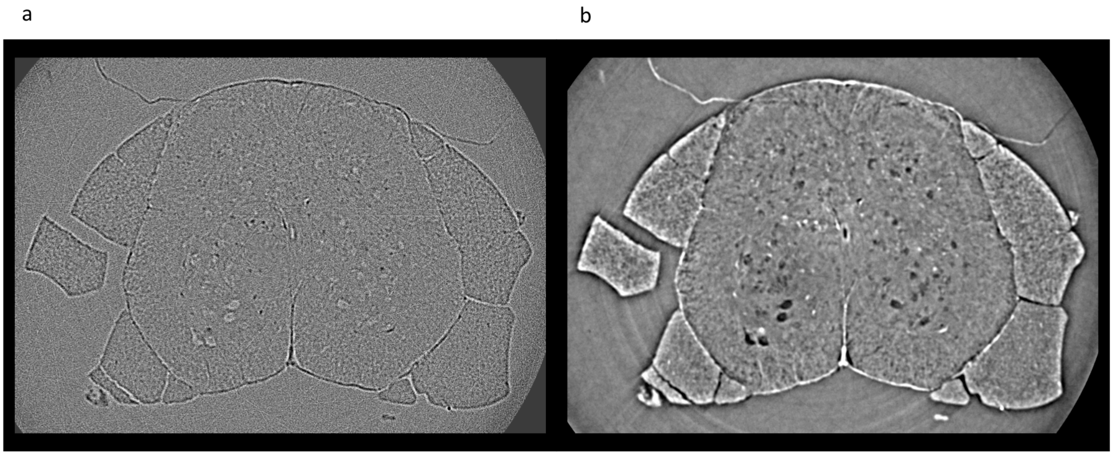

Figure 1S: Axial slice of naïve mouse spinal cord. The image in a) is the result obtained just after tomographic reconstruction from the measured projections. The image in b) is the result obtained after application of the phase retrieval process. The differences between the two images are evident. The different details with different density (different grey levels), both in the white and in the grey matter, appear well visible in b) but are not appreciable in a).

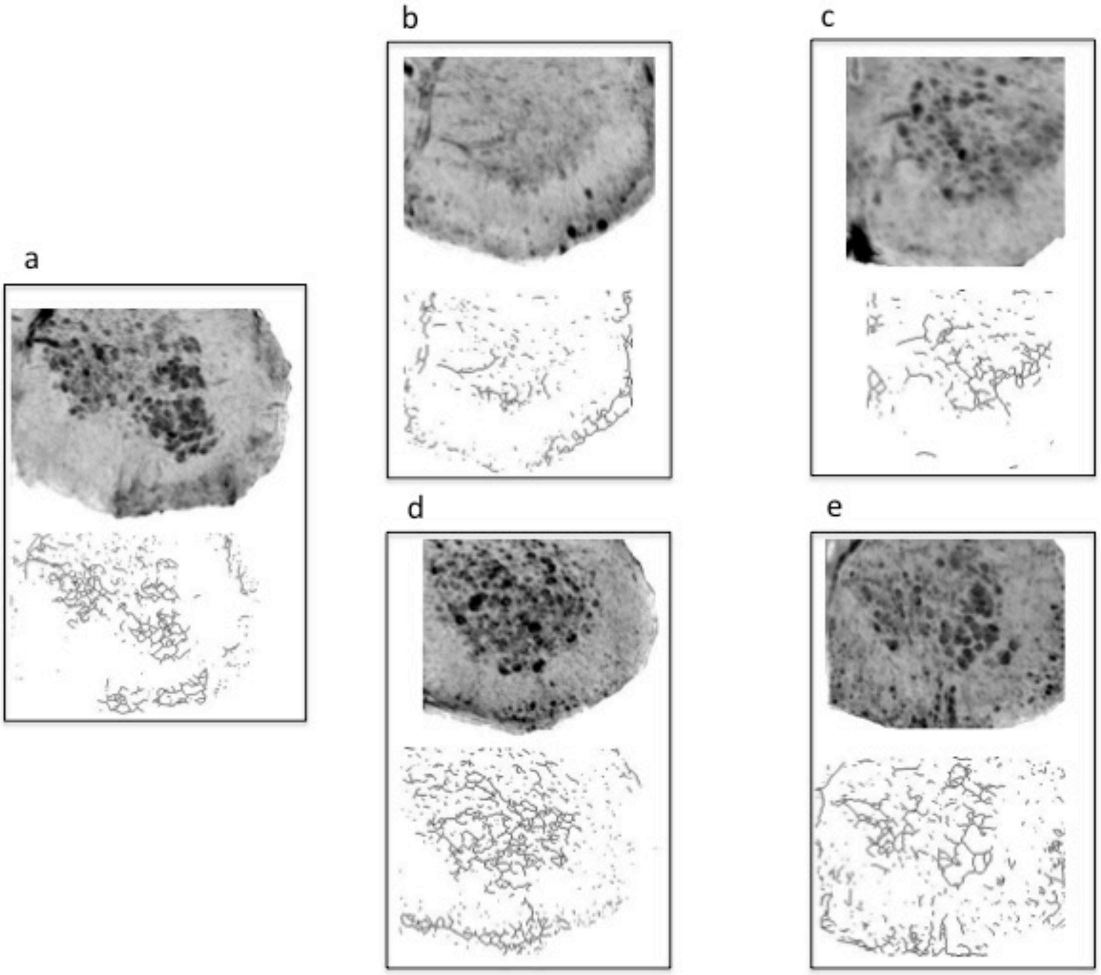

Figure 2S: Segmentation for the quantification of vascular alteration at the initial phase of EAE. Axial projections of XPCT images of the spinal cord in naïve mouse (a), in EAE-affected mice at 1 dpo (b) and 5 dpo (c), and in MSC-treated EAE-affected mice at the corresponding time points (d, e). The bottom part of each figure shows the segmentation of the vessels necessary for the calculations of the number of branches.

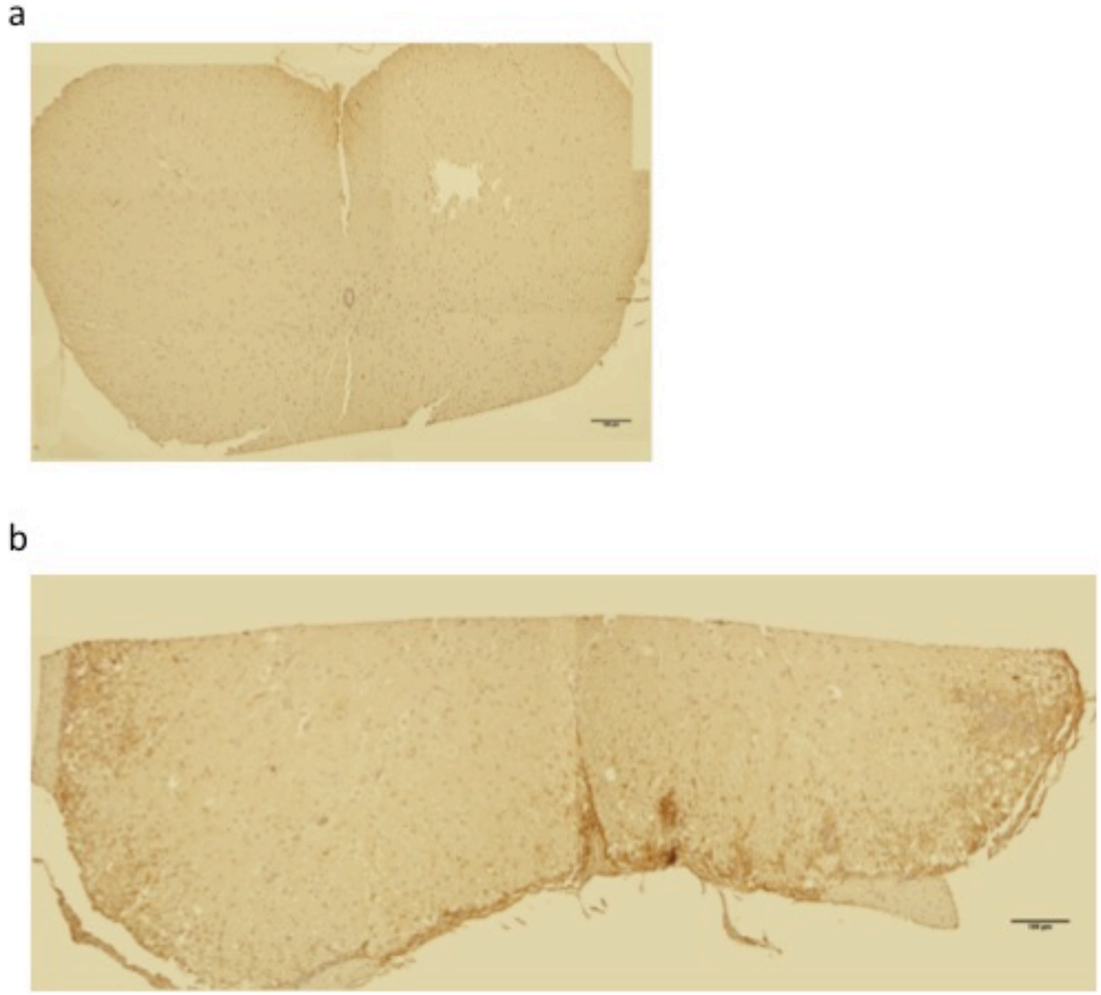

Figure 3S: Increased staining for CD31 in EAE. Immunohistochemical analysis of naïve (a) and EAE (b; sample obtained at 5 dpo) spinal cord samples. Spinal cord slices were stained with a monoclonal rat anti-mouse CD31 antibody (clone SZ31, Dianova, Germany) developed with biotinylated goat anti-polyvalent antibody itself revealed with streptavidin peroxidase (Ultravision Plus Large volume detection system, Thermo Fisher Scientific, USA), as per manufacturer’s instructions.

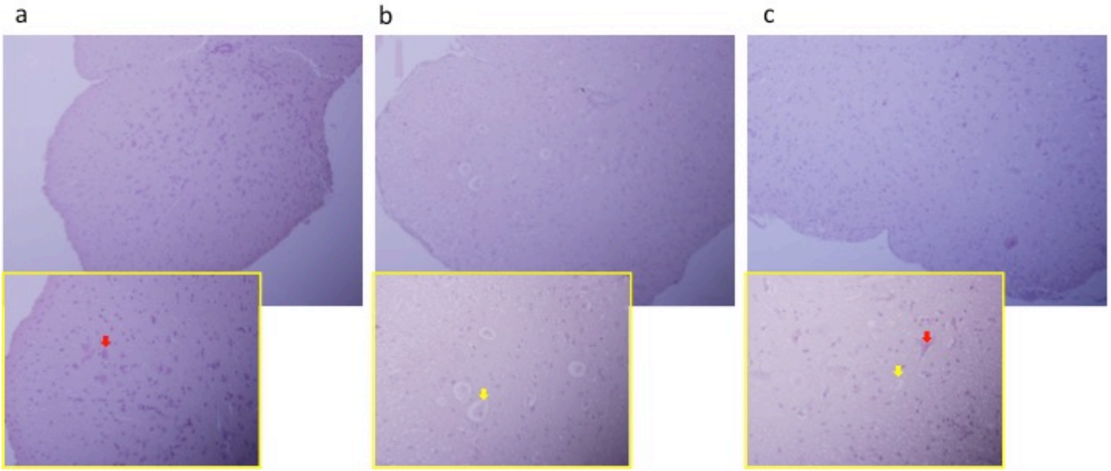

Figure 4S: Histological analysis of spinal cord samples from naïve and untreated and MSC-treated EAE-affected mice suggests that MSC treatment decreases the incidence of pyknotic neurons in EAE. Yellow arrows indicate pyknotic cells; red arrows indicate ‘normal’ cells. Scale bar: 100 μm.

References

1. Weitkamp, T., Haas, D., Wegrzynek, D. & Rack, A. ANKAphase: software for single-distance phase retrieval from inline X-ray phase-contrast radiographs. *J. Synchrotron Radiat.* **18**, 617-629 (2011).

2. Paganin, D., Gureyev, T. E., Pavlov, K. M., Lewis, R. A. & Kitchen, M. Phase retrieval using coherent imaging systems with linear transfer functions. *Opt. Commun.* **234**, 87-105 (2004).
